# Identification of 4′-Demethyl-3,9-dihydroeucomin as a Bitter-Masking Compound from the Resin of *Daemonorops draco*

**DOI:** 10.1021/acs.jafc.4c04583

**Published:** 2024-09-15

**Authors:** Sonja Sterneder, Joachim Seitz, Johannes Kiefl, Eric Rottmann, Margit Liebig, Maria Blings, Stephan Seilwind, Yijun Zhou, Jianbing Wei, Haifeng Guan, Qianjin Zhu, Johanna Kreißl, Kai Lamottke, Jakob P. Ley, Veronika Somoza

**Affiliations:** † Department of Physiological Chemistry, Faculty of Chemistry, University of Vienna, 1090 Vienna, Austria; ‡ Vienna Doctoral School in Chemistry (DoSChem), Faculty of Chemistry, University of Vienna, 1090 Vienna, Austria; § Leibniz Institute for Food Systems Biology, Technical University of Munich, 85354 Freising, Germany; ∥ Chair of Nutritional Systems Biology, TUM School of Life Sciences, Technical University of Munich, 85354 Freising, Germany; ⊥ Symrise AG, 37603 Holzminden, Germany; # Bicoll Biotechnology (Shanghai) Co., Ltd., 201203 Pudong, China; ∇ Bicoll GmbH, 82152 Planegg/Martinsried, Germany

**Keywords:** bitter-masking compound, 4′-demethyl-3,9-dihydroeucomin, Daemonorops draco, cellular bitter response, activity-guided approach

## Abstract

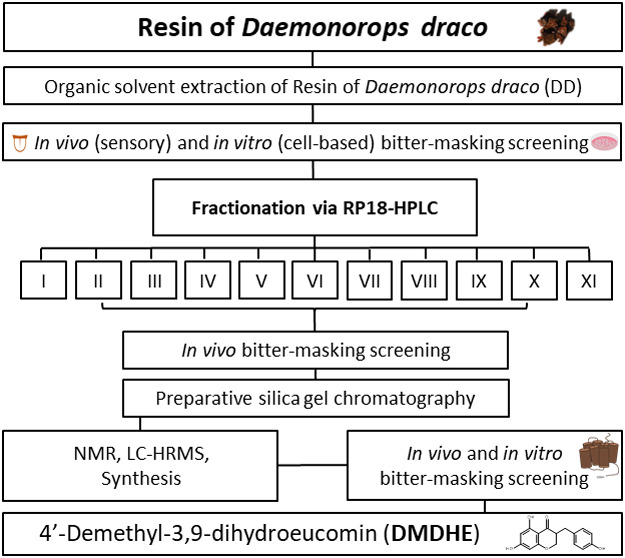

Masking the bitter taste of foods is one of the key strategies to improve their taste and palatability, particularly in the context of clean labeling, where natural compounds are preferred. Despite the demand, the availability of natural bitter-masking compounds remains limited. Here, we identified the bitter-masking compound 4′-demethyl-3,9-dihydroeucomin (**DMDHE**) isolated from the resin of *Daemonorops draco* by means of an activity-guided in vivo (sensory bitterness rating of quinine) and in vitro (cell-based bitter response assays) approach. First, a mean bitter-masking effect of −29.6 ± 6.30% on the bitterness perceived from quinine [10 ppm] was demonstrated for an organic solvent extract of the resin of *D. draco* (= DD [500 ppm]) in a sensory trial. The results were verified in a cell-based bitter assay in which the bitter taste receptor (TAS2R)-dependent proton secretion serves as an outcome measure of the cellular bitter response in parietal HGT-1 cells. By means of preparative RP-18 high-performance liquid chromatography (HPLC) analysis combined with activity-guided sensory evaluations, the most potent bitter-masking fractions were identified. Subsequent quantitative liquid chromatography/high-resolution mass spectrometry/charged aerosol detection/ultraviolet (LC-HRMS/CAD/UV), NMR analysis, followed by gram-scale synthesis, led to the characterization of **DMDHE** as bitter-masking homoisoflavanone. **DMDHE** decreased the sensory bitterness of quinine by 14.8 ± 5.00%. Functional involvement of TAS2R14 was demonstrated by means of a CRISPR-Cas9 approach, which revealed a reduction of the **DMDHE**-evoked bitter-masking effect by 40.4 ± 9.32% in HGT-1 *TAS2R14ko* versus HGT-1 wt cells.

## Introduction

Bitter taste masking is a technique widely used in food, beverage, and pharmaceutical industries to cover up or reduce the perception of bitter flavors. It involves employing various methods to either alter or block the bitter taste sensation while maintaining the desired characteristics of the product. Major strategies applied for bitter taste masking are based on either physical or chemical approaches. Physical approaches involve encapsulation, by which the bitter substance is coated with^1^ or encapsulated within^2^ another substance to prevent it from being immediately perceived by bitter taste receptors (TAS2Rs) located in the taste buds of our tongue. Chemical bitter masking involves (i) altering the chemical structure of the bitter compound to modulate its binding affinity to TAS2Rs^3^ and thereby its bitterness while maintaining its functional properties. Moreover, (ii) adding other strong flavors that can overpower or distract from the bitterness by either antagonizing TAS2Rs and/or activating other taste receptors, e.g., the sweet taste receptor, by adding sweeteners, can be used to balance or hide the bitterness.

One of the current challenges in bittermasking lies within clean labeling, which is in high demand by consumers and requires careful selection and formulation of ingredients to ensure a product’s integrity. Clean labeling refers to the practice of using simpler formulations and more natural ingredients in food and beverage products, usually avoiding artificial additives, preservatives, and complex chemical names on the ingredient list. When it comes to bitter masking in clean labeling, the challenge lies in finding natural and recognizable ingredients or techniques to effectively mask bitterness without compromising either the clean label aspect or the product palatability.

In this work, we describe the identification of a novel bitter-masking compound from the resin of *Daemonorops draco*, a species of palm native to Southeast Asia. This plant is commonly known as the “Dragon’s Blood Palm” because of its vivid red resin, often referred to as “dragon’s blood,” which has been used for centuries in Traditional Chinese medicine for various medicinal purposes, making use of its, e.g., anti-inflammatory^4^ or anticancerogenic effects.^5^ Flavonoid-like structures from a related source of “dragon’s blood,” *Dracaena cinnabari*, could be identified as potent taste-modifying substances (data not published). Due to the very complex chemical composition of those resins, an activity-guided fractionation based on a cell-based assay combined with human sensory testing was performed. The experimental approach applied for the identification of a novel bitter-masking compound from *D. draco* is based on the molecular mechanism of TAS2Rs: TAS2Rs were targeted by known agonists in sensory experiments and in a surrogate cell model and studied for their sensory and cellular response, respectively, with/without coexposure of an organic solvent extract of *D. draco* (DD) and purified constituents thereof. The use of a surrogate model allowed the identification of one of the TAS2Rs chiefly involved in the bitter-masking effect of the *D. draco* constituent.

The surrogate cell model (human gastric tumor cell line-1, HGT-1) established previously is derived from a poorly differentiated adenocarcinoma^6^ and is well-established as a model for studies of TAS2R-dependent mechanisms of gastric acid secretion.^6,7^ In previous studies of our group, we demonstrated HGT-1 cells to express the same pattern of TAS2Rs as primary cells of the human tongue^8,9^ and gastric antrum,^9^ with TAS2R14 showing the highest level of gene expression.^9^ TAS2R14 is the most broadly tuned TAS2R, with the highest number of reported agonists (>200)^10,11^ including, e.g., quinine,^12^ caffeine,^12^ and quercetin.^13^ TAS2R14 also represents the TAS2R for which the highest number of identified antagonists is known,^14^ e.g., the polyphenols 6-methoxyflavanone^15^ and homoeriodictyol (HED).^16^

When treated with a bitter-tasting compound, e.g., caffeine, HGT-1 cells respond by the secretion of protons, thereby lowering their intracellular proton concentration. This response is the result of the bitter-tasting compound activating one or more TAS2Rs, which triggers intracellular signaling cascades that lead to proton secretion.^9,17−19^ The number of protons secreted results in a drop of intracellular pH, which is quantitated by means of a pH-sensitive fluorescent dye, whereby a high proton secretion correlates with a strong bitter response and vice versa.^9,18,20^

**Figure 1:**
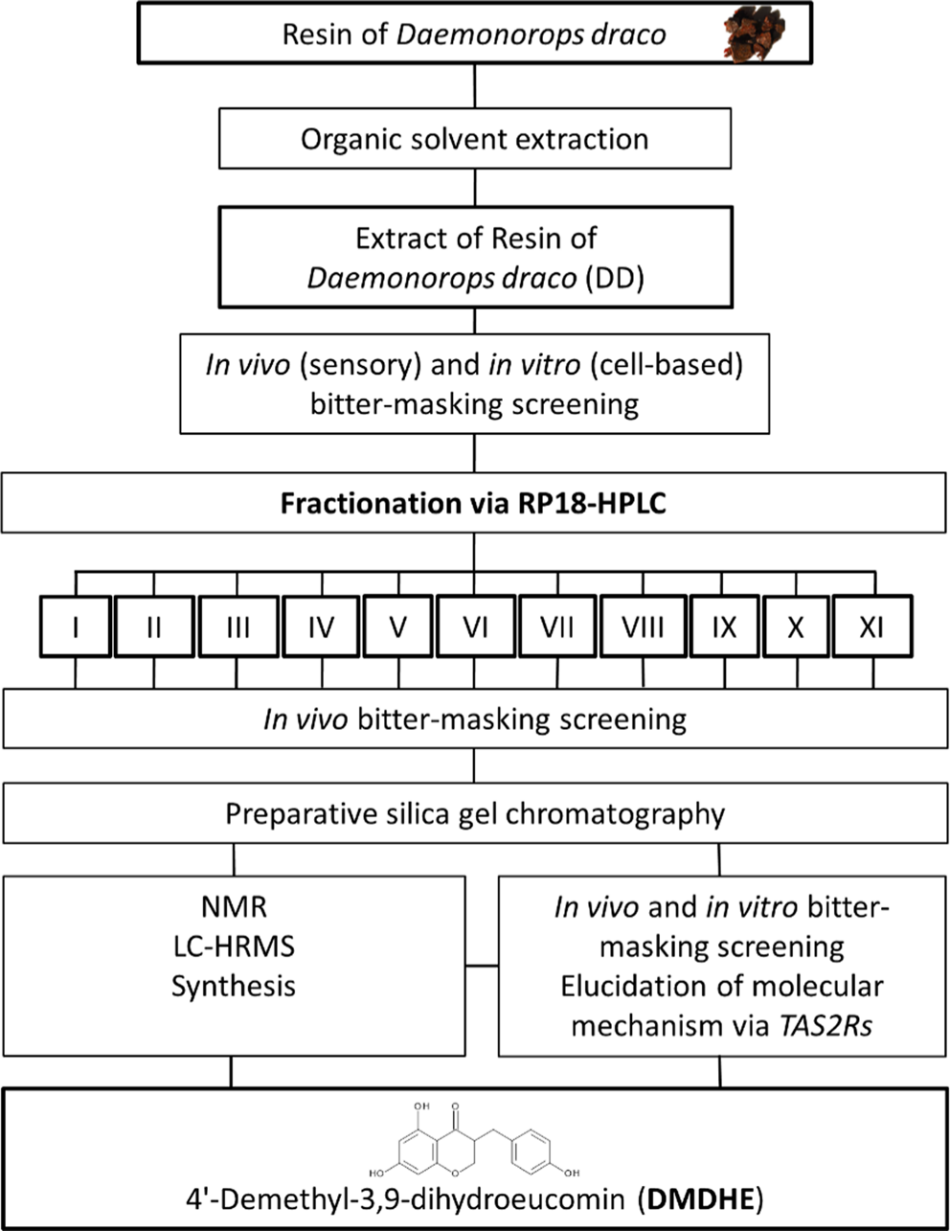
Experimental approach.

The suitability of this cell model for the identification of bitter-tasting and bitter-taste-modulating compounds has been reported in various studies during the past decade.^9,17−19,21−23^ In this work, we combined the HGT-1 cell-based bitter response assay with analytical separation techniques and human sensory trials in an activity-guided approach to identify a novel bitter-masking plant constituent for use in foods and beverages that meet the standards of clean labeling. A brief overview of the experimental approach is shown in Figure 1.

## Materials and Methods

### Chemicals

Deionized water was prepared by an Arium Mini UV laboratory water system (Sartorius, Göttingen, Germany). The resin of *D. draco* (100 g) was obtained by Bicoll. Dulbecco’s Modified Eagle’s Medium (DMEM), trypsin, glutamine, penicillin/streptomycin, histamine (purity ≥97%), and primer oligonucleotides were purchased from Sigma-Aldrich. 1,5-Carboxy-seminaphtorhodafluor acetoxymethyl ester (SNARF-1 AM), nigericin, fetal bovine serum (FBS), and the reagents for gene editing, TrueCut Cas9 Protein v2, Lipofectamine CRISPRMAX Cas9 Transfection Reagent, TrueGuide Synthetic sgRNA (positive control: HPRT1, negative control: nontarget 1 and target: TAS2R14), GeneArt Genomic Cleavage Detection Kit, Opti-MEM I Reduced-Serum Medium, and TE buffer pH = 8.0, were obtained from Thermo Fisher Scientific. The RT-qPCR material was purchased from Thermo Fischer Scientific. Phosphate-buffered saline 1× (PBS) was obtained from Biozym Biotech Trading GmbH (Austria). 3-(4,5-Dimethylthiazol-2-yl)-2,5-diphenyltetrazolium bromide (MTT) for the MTT assay was obtained from Carl Roth GmbH + Co., KG (Austria). For cell culture studies, quinine-HCl (Combi-Blocks) was used and is mentioned in the text as quinine. Compound 4′-demethyl-3,9-dihydroeucomin (**DMDHE**, CAS 107585–77–3, purity ≥98%) was obtained from BOC Science and synthesized by Bicoll. LC-MS grade acetonitrile and formic acid were purchased from Riedel-de Haen (Seelze, Germany).

### Preparation of the *D. draco* Extract

The resin of the fruit of *D. draco* (“Xue Jie”) was collected in Yunnan province, China (NAGOYA Protocol approved). An amount of 300 g of the resin was ground into powder, extracted with 95% ethanol, and filtrated to remove particles. After ethanol was removed, the residue was solubilized in dichloromethane, loaded onto a solid phase material, and consecutively eluted with *n*-hexane and ethyl acetate. The resulting organic solvent extract of *D. draco* was named DD.

### Preparative Column Chromatography

Preparative chromatography of DD was performed using an RP-18-HPLC system (P2.1L, Knauer, Germany) with a column oven at 20 °C (Jetstream), a fraction collector (Foxy R1, Teledyne ISCO), and a UV detector (S2600, Knauer, Germany) equipped with a silica 5 μm 100 Å 250 mm × 21.2 mm (Luna, AXIA, Phenomenex) column using a hexane/ethyl acetate gradient. Hexane concentration was decreased within 18 min from 55 to 50%, further decreased to 40% within another 7 min, further decreased to 5% within another 3 min, and held for 6 min at 5% hexane/95% ethyl acetate.

### Paired Comparison Sensory Test

A trained panel was given two samples per test, with the bitter-tasting substance quinine [10 ppm] dissolved in water on one hand and the potential bitter-masking extract, fractions, or compound on the other hand. In preliminary studies, the quinine concentrations used by Ley et al.^24^ were tested, but for this work, we made small adjustments to find a concentration that was above the bitter taste threshold^25^ and still acceptable. The test concentration of 10 ppm is still below the set tested by Tenney et al.^26^

In the first set of the sensory tests, a concentration of 500 ppm of DD was based on initial HPLC analysis data, which revealed flavonoid-like structures between approximately 5 and 20% (UV, data not shown). According to this data, a concentration of 500 ppm of DD was considered equivalent to a concentration of putatively bitter-masking flavonoids of approximately 75–100 ppm. This target concentration was chosen, since previous studies demonstrated an effective bitter-masking concentration of 100 ppm for flavanones from *Herba santa*.^24^ Consequently, a concentration of 100 ppm of **DMDHE** was chosen in the second set of sensory experiments.

With respect to toxicological considerations, the dragon’s blood extract had been listed in FEMA 2404 before the current study, with a maximum dosage of 1000 ppm. Additionally, for **DHMDE**, in silico toxicology was estimated using DEREK (Derek KB 2020 1.0, Lhasa Limited, Leeds, Yorkshire, U.K.). Since a chromosome aberration match in mammals (523 alkylphenols) was alerted, a professional toxicologist performed a cross-read to a structurally similar compound, phloretin, which was evaluated without any genotoxic potential/concern by the Joint FAO/WHO Expert Committee on Food Additives (JECFA) in 2011. According to this cross-read, the toxicologist approved sensory testing by the sip and spit method up to a concentration of 100 ppm (three tests per day per person with a maximum exposure of 5 μg per kg body weight and day to be expected, thereby not exceeding the limit for Cramer Class III compounds of 90 μg kg per body weight and day).

During the tests, the panelists wear nose-clips to exclude any olfactory impressions. Samples were served with a 3-digit code in a sensory room with yellow-red light. Panelists were asked to rate the bitter intensity on a scale of 0–10. Data were recorded and statistically elaborated with Eyequestion 4, Logic8 B.V.

### Chemical Characterization of Extracts, Fractions, and Compounds

Extracts and fractions were characterized by liquid chromatography (Acquity UPLC system, Waters) coupled with high-resolution mass spectrometry (microTOFQII, Bruker) and charged aerosol detection (Corona Veo, Thermo Fisher Scientific, Germany). Mass spectra were acquired in ESI+ and ESI– ionization modes and a scan range of 50–1600 Da. Chromatographic separation was carried out on a C18 column (Kinetex, 100 mm × 2.1 mm, 1.7 μm; Phenomenex) at a temperature of +50 °C and a flow rate of 0.55 mL/min using an acetonitrile/water gradient. Two microliters were injected, and the gradient started with 100% water containing 0.1% formic acid, increasing to 95.0% acetonitrile within 22 min. This concentration was maintained for 5 min. Peak identity was confirmed either by co-chromatography (LC-HRMS/CAD/UV) of the reference compounds or by NMR analysis. ^1^H, ^13^C, COSY, HSQC, and HMBC measurements were performed on a Bruker Avance-III 600 MHz spectrometer Progidy-BBO-Probe and a Bruker Avance-III 400 MHz spectrometer BBO-Probe at 298 K (Bruker, Rheinstetten, Germany). Data processing was performed with MNova (version 9, Mestrelab Research, Santiago de Compostela, Spain) or Topspin (version 3.2, Bruker, Rheinstetten, Germany). Spectra were recorded in DMSO-*d*_6_ and referenced to tetramethylsilane. Purity was calculated either as the area percent value relative to all peaks detected by charged aerosol detection or as the area percent value relative to all ^1^H signals detected by NMR.

### Cell Culture

HGT-1 cells were attained from C. Laboisse (Laboratory of Pathological Anatomy, Nantes, Frances). Cultivation of HGT-1 cells was performed under standard conditions (in DMEM (with 10% FBS, 4 mM glutamine, and 1% penicillin/streptomycin) at 37 °C, 95% humidity, and 5% CO_2_) and seeded 24 h prior to the measurement with a density of 100,000 cells per well in 96-well plates, of which transparent ones were used for the cell viability assay (MTT assay) and black ones for the PS assay to determine the IPX. For gene expression analysis, 700,000 cells per well were seeded in a transparent 6-well plate 24 h before incubation with the substances of interest.

### Cell Viability

The viability of the HGT-1 cells was investigated via the MTT test (3-(4,5-dimethylthiazol-2-yl)-2,5-diphenyltetrazolium bromide) to exclude negative metabolic effects of the test fractions and compounds, as described before.^23^ For this purpose, the cells were incubated for 15 min according to the incubation time for gene expression and intracellular pH analysis. Treatment of the cells was performed with DMSO (0.1%) as solvent control, quinine [10 ppm] w/o DD [100 ppm] or **DMDHE** [100 ppm]. After removal of the test fractions/compounds, 100 μL of MTT solution (0.83 mg/mL in DMEM) were added to each well, and absorbance of the treated cells was read out at 570 nm, with a reference wavelength 650 nm, using an Infinite 200 Pro Plate Reader (Tecan, Männderdorf, Switzerland). Cell viability of the treated cells was calculated relative to nontreated controls (buffer-treated = 100%) and displayed in percent.

### Determination of the Intracellular pH in HGT-1 Cells

Quantitation of the intracellular proton concentration was performed using the pH-sensitive fluorescence dye SNARF-1 AM, as described before.^9^ Briefly, the HGT-1 cells were cultivated as described above and washed with 100 μL of Krebs–Ringer–HEPES buffer (KRHB; 10 mM 4-(2-hydroxyethyl)-1-piperazineethane-sulfonic acid (HEPES), 11.7 mM d-glucose, 4.7 mM KCl, 130 mM NaCl, 1.3 mM CaCl_2_, 1.2 mM MgSO_4_, and 1.2 mM KH_2_PO_4_, adjusted to a pH of 7.4 with 3M KOH) per well. After staining the cells with 3 μM 1,5-carboxy-seminaphtorhodafluor acetoxymethyl ester (SNARF-1 AM) for 35 min, two washing steps with 100 μL of KRHB/well were performed. Afterward, the cells were treated with the samples (quinine [10 ppm] w/o DD [100 ppm], **DMDHE** [100 ppm]), KRHB + DMSO [0.1%] as solvent control, or histamine [1 mM] as a positive control. The fluorescence measurement was performed using a Flexstation 2 device (Molecular Devices, California) at 580 and 640 nm emission after excitation at 488 nm. Intracellular proton concentration was quantitated by means of a pH calibration curve, followed by calculating the percent difference between untreated (control = KRHB + DMSO [0.1%]) versus treated cells.^9^

### TAS2R Gene Expression

Changes in TAS2R mRNA transcript levels were evaluated after incubation of the HGT-1 cells with quinine [10 ppm] w/o DD [100 ppm] diluted in DMSO (0.1% fc on the cells) for 15 min. mRNA was isolated using the peqGOLD Total RNA Kit (VWR Life Science, Germany), and mRNA quality and quantity were determined spectrophotometrically at 260 and 280 nm with a nanoQuant plate for the Infinite 200 Pro Plate Reader (Tecan, Männderdorf, Switzerland). RNA samples that passed quality control, namely, OD between 2.0 and 2.2, were used for cDNA synthesis using the High-Capacity cDNA Reverse Transcription Kit (Applied Biosystems, Thermo Fisher Scientific, Vienna, Austria). Quantitative PCR was carried out using the StepOnePlus Real-Time PCR system (Applied Biosystems, Thermo Fisher Scientific, Vienna, Austria) with the following protocol: activation (95 °C for 20 s), denaturation (95 °C for 3 s), and annealing (60 °C for 30 s) for 45 cycles. Primers for the investigated genes of the bitter taste receptors (*TAS2R4*, *TAS2R7*, *TAS2R10*, *TAS2R14*, *TAS2R31*, *TAS2R39*, *TAS2R40*, *TAS2R43*, *TAS2R46*) and the housekeeping genes (*GAPDH*, *TBP*, and *PPIA*) were designed and validated previously^9^ (Table S2). All bitter taste receptors known to be targeted by quinine were selected for the investigation. To determine the hypothetical mRNA starting concentrations, RT-qPCR data were analyzed with LinRegPCR.

### Transient CRISPR-Cas9 Transfection

HGT-1 cells were seeded in 96-well plates with a density of 15,000 cells per well and settled for 24 h. Transfection reagent, Invitrogen TrueCut Cas9 Protein v2 (Thermo Fisher Scientific), was mixed with the TrueGuide Synthetic sgRNA (*TAS2R14* (U*G*A* UCGGAUCCUCACUGCUU + modified Scaffold), negative control or positive control), Lipofectamine Cas9 Plus Reagent (Thermo Fisher Scientific), and Opti-MEM I Medium (Thermo Fisher Scientific). In the second step, the Lipofectamine CRISPRMAX Reagent, diluted in Opti-MEM I Medium (Thermo Fisher Scientific), was added, and after an incubation time, the combination of both was applied onto the cells and incubated for 48 h. The transfection efficacy of a mean of 20% was analyzed with the Genomic Cleavage Detection Kit (Thermo Fisher Scientific) according to the manufacturer’s protocol, as published previously.^17^ The primers shown in Table S3 were used for the genomic cleavage detection analysis by applying the following temperature protocol: 95 °C/10 min, 40 cycles of 95 °C/30 s, 55 °C/30 s, 72 °C/30 s, and finally 72 °C for 7 min. A genomic cleavage of 19% was detected. With the transfected HGT-1 cells (*TAS2R14ko*), a cellular bitter response assay was performed according to the above-mentioned protocol.

### Statistics

Data analysis was conducted with Excel (Microsoft) and GraphPad Prism (Dotmatics, California). Data are shown as the mean ± standard error of the mean (SEM) unless stated otherwise. Numbers of replicates for each experiment are stated in the Results and Discussion section but were at least *n* = 3. Nonconfident values were identified using the Nalimov test. All data were tested for normality and equal variances. According to the data distribution and number of groups, Student’s *t*-test or one-way ANOVA with adequate posthoc tests was conducted.

## Results and Discussion

Masking the bitter taste is one of the key strategies to improve the taste, palatability, and overall appeal of foods, food formulations, or medicinal products. Moreover, existing product portfolios could be expanded by creating new products or formulations that might have been avoided due to unpleasant taste profiles.

In the context of clean labeling, finding bitter-masking compounds from plant sources allows for addressing taste challenges in product formulations while maintaining transparency and meeting consumer preferences for natural, easily understandable ingredients. Current research aims to discover and isolate effective bitter-masking compounds from plant sources, since the number of these compounds from natural sources is still limited, given the variety of bitter-tasting foods and formulations thereof. Here, we identified 4′-demethyl-3,9-dihydroeucomin (**DMDHE**) as a bitter-masking compound from an organic solvent extract of the resin of *D. draco* (DD). The experimental approach started with sensory studies by which the masking effect of DD on the bitter taste perceived from quinine was demonstrated. In search for the key bitter-masking constituent, DD was subjected to an activity-guided fractionation, in which analytical separation methods were combined with a cell-based bitter response assay and in parallel an in vivo sensory assay. Finally, the isolated and identified key active compound, **DMDHE**, was validated for its bitter-masking properties in a human sensory trial again. The underlying molecular mechanism was demonstrated by gene editing targeting bitter taste receptor *TAS2R14* by means of the CRISPR-Cas9 knockout approach.

### Bitter-Masking Effect of DD In Vivo and In Vitro

DD was tested in a pairwise comparison sensory test for its bitter-masking effect on the bitterness perceived from quinine. Quinine is commonly used to provide the bitterness of tonic water and is often applied as a reference compound in bitter testing sensory trials in concentrations ranging from 3^25^ to 67 ppm.^26^

**Figure 2:**
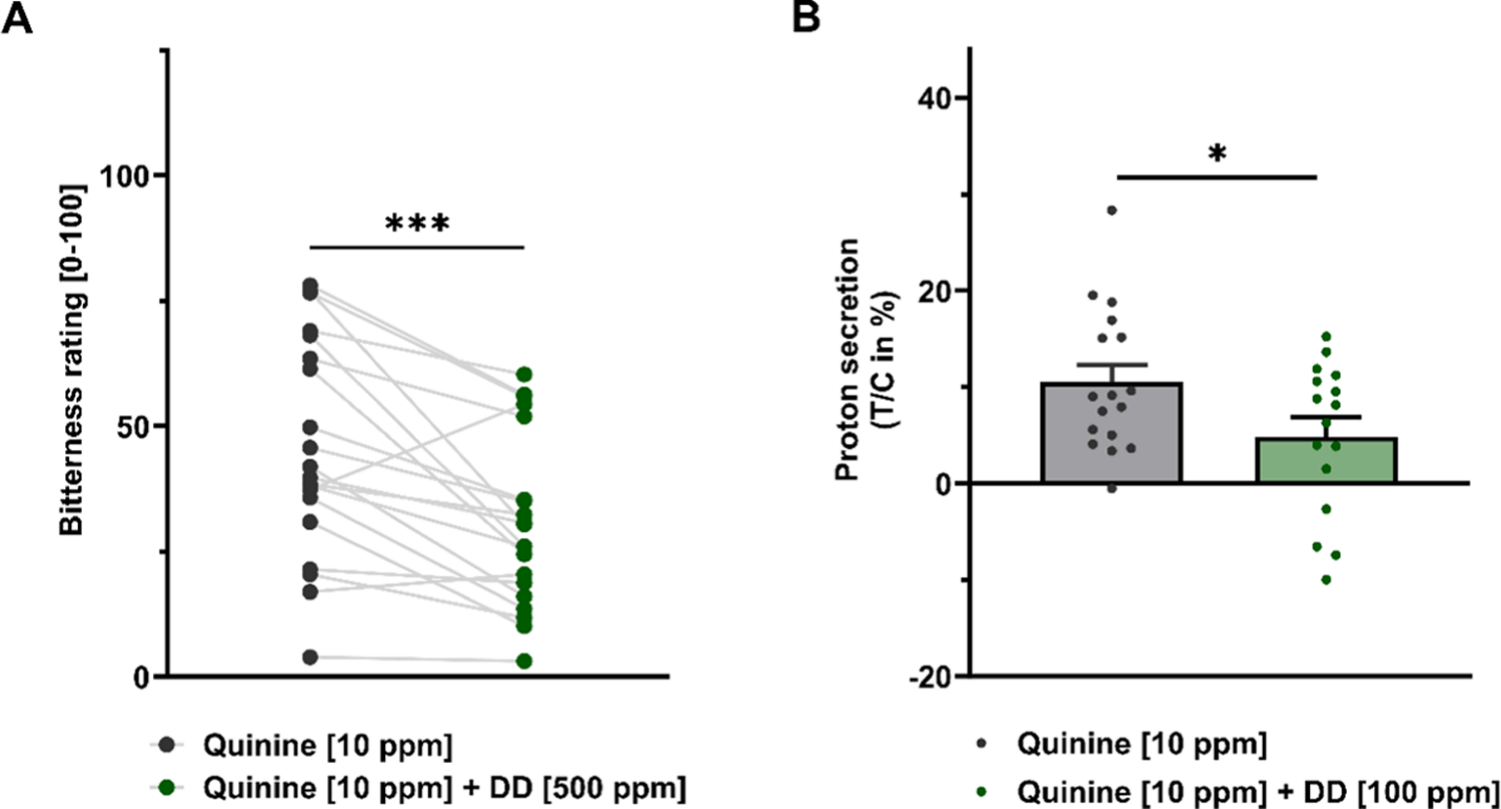
(A) Bitterness rating [0 not bitter–100 very bitter] of quinine [10 ppm] w/o addition of the DD extract [500 ppm] (*n* = 20). Statistical analysis: Paired Student’s *t*-test, significance is indicated with ***p ≤ 0.001. (B) Impact of quinine [10 ppm] w/o the addition of the DD extract [100 ppm] on the percent increase in proton secretion in HGT-1 cells compared to controls (nontreated control cells = 0) after a 10 min treatment time; statistical analysis: Unpaired Student’s *t*-test, significance is indicated with *p ≤ 0.05. Data are shown as mean ± SEM, *n* = 3, t.r. = 4–6.

When the panelists compared the bitterness of quinine [10 ppm] with a combination of quinine [10 ppm] and DD [500 ppm], the bitterness perceived of quinine was reduced by a mean of −29.6 ± 6.30% (*p* ≤ 0.001, Figure 2A).

Getting a first insight into the molecular mechanisms of the bitter-taste-modulating activity of DD, the established surrogate HGT-1 cell model^9,18,20,22,23^ was studied for its cellular bitter response via functional involvement of TAS2Rs. In this cell model, an increased, TAS2R-dependent, cellular proton secretion is followed by a decreased intracellular proton concentration [H^+^]. In accordance with the concentrations tested in the sensory trial, HGT-1 cells were treated with a concentration of 10 ppm (27.7 μM) quinine, which reduced the intracellular proton concentration compared to control cells ([H^+^]_control_: 2.06 × 10^–8^ ± 7.06 × 10^–10^ to [H^+^]_quinine_: 1.84 × 10^–8^ ± 3.61 × 10^–9^; *p* < 0.01) by a mean of 10.5% as a consequence of the increased secretion of protons after a 10 min treatment. Coincubation of quinine [10 ppm] and DD [100 ppm] ameliorated the quinine-evoked increase in proton secretion by a mean of −53.5% (from +10.5 ± 29.8% for quinine to +4.87 ± 30.5% for quinine + DD; *p* < 0.05; Figure 2B). This finding indicates a bitter-masking effect of DD in the HGT-1 cell model, which is consistent with the sensory results. Noteworthy, due to a limited solubility of DD in the solvent used for the cell culture experiments (DMSO 0.1% fc on the cells), the DD concentration of 100 ppm applied here was five times lower than the 500 ppm tested in the sensory trial. However, a bitter-masking effect for DD was demonstrated in both test systems, thereby underlining the suitability of the previously established HGT-1 surrogate model for the identification of bitter-tasting and bitter-masking compounds.^20,23^

The involvement of TAS2Rs in the bitter-masking effect of DD was studied by quantitating their mRNA expression levels after treatment with quinine w/o coincubation of DD. After adjusting the effect of DD to the quinine-evoked TAS2R mRNA regulation (set = 1), mRNA levels of *TAS2R4*, *TAS2R14*, and *TAS2R43* were reduced by a mean fold change of 0.81 ± 0.02, 0.66 ± 0.04, and 0.66 ± 0.06 (*p* < 0.05 for all) (Table 1). Since *TAS2R14* is known to be the most broadly tuned TAS2R, agonized by the highest number of more than 200 compounds known so far,^10,11,14^*TAS2R14* was selected for the next set of in vitro experiments, which aimed the identification of the molecular mechanisms of the key bitter-taste-masking DD constituent.

**Table 1:** mRNA Expression of Selected TAS2Rs in HGT-1 Wild-Type Cells after Incubation of the Cells with Quinine [10 ppm] and DD [100 ppm] for 15 min^a^

**gene**	**fold change (quinine vs controls)**	**fold change(quinine + DD)**	***p*-value**
*TAS2R4*	1.00 ± 0.05	0.81 ± 0.02	0.026
*TAS2R7*	1.00 ± 0.15	1.14 ± 0.30	0.663
*TAS2R10*	1.00 ± 0.11	0.72 ± 0.08	0.104
*TAS2R14*	1.00 ± 0.10	0.66 ± 0.03	0.030
*TAS2R31*	1.00 ± 0.08	0.84 ± 0.04	0.186
*TAS2R39*	1.00 ± 0.07	0.83 ± 0.09	0.159
*TAS2R40*	1.00 ± 0.15	0.67 ± 0.08	0.143
*TAS2R43*	1.00 ± 0.09	0.66 ± 0.06	0.016
*TAS2R46*	1.00 ± 0.05	0.92 ± 0.06	0.386

a Fold changes of mRNA expression were adjusted to the response to a concentration of 10 ppm quinine (= 1), *n* = 4, t.r. = 3. Statistics: Unpaired Student’s *t*-test between quinine-treated HGT-1 cells versus quinine + DD-treated cells.

### Identification of Bitter-Masking Fractions of DD

The bitter-masking effect of DD on quinine encouraged the identification of a bitter-masking constituent thereof by means of an activity-guided fractionation approach based on bitter-masking screening in vivo and in vitro.

In the first step, DD was separated by preparative RP-18 HPLC-UV to obtain 11 fractions (**I**–**XI**, Figure 1), which were subjected to pairwise comparison sensory tests to identify their bitter-masking property on 10 ppm quinine (Table 2). Fractions **V** and **IX** showed the highest bitter-masking effect, here with a mean reduction of the bitterness perceived from quinine by −24.2 ± 5.19% (*p* ≤ 0.001) and −17.4 ± 6.50% (*p* ≤ 0.001), respectively. Notably, the concentration of the tested fractions shown in Table 2 varies between 30 and 50 ppm due to differences in solubility or limited available quantity (yield). The differences in the panel sizes from 10 to 20 volunteers are based on varying drop-out rates. We admit that this differing number of panelists is a limitation of our work, since the statistical power might not have been sufficiently high enough to identify more fractions containing bitter-masking flavonoids. Therefore, we cannot exclude the existence of additional bitter-masking compounds in other fractions, e.g., fractions **II**, **IV**, and **VIII**.

**Table 2:** Sensory Study^a^

**DD RP-18-HPLC fractions**	**dosage [ppm]**	**panel (*n*)**	**mean bitter-masking effect on quinine [%]**	***t*-test(*p*-value)**
**I**	50	20	–13.7 ± 6.48	0.053
**II**	30	12	–21.9 ± 10.9	0.075
**III**	50	20	–25.1 ± 4.77	0.001
**IV**	30	20	–14.9 ± 6.30	0.029
**V**	50	20	–24.2 ± 5.19	0.000
**VI**	30	12	–8.87 ± 9.09	0.253
**VII**	30	10	–19.2 ± 7.55	0.062
**VIII**	30	10	–20.5 ± 5.51	0.007
**IX**	50	20	–17.4 ± 6.50	0.004
**X**	30	20	–20.6 ± 6.62	0.010
**XI**	30	20	–0.37 ± 5.75	0.981

a Pairwise comparison sensory test of HPLC fractions of DD w/o quinine [10 ppm].

### Identification of **DMDHE** in DD Fractions

**Figure 3:**
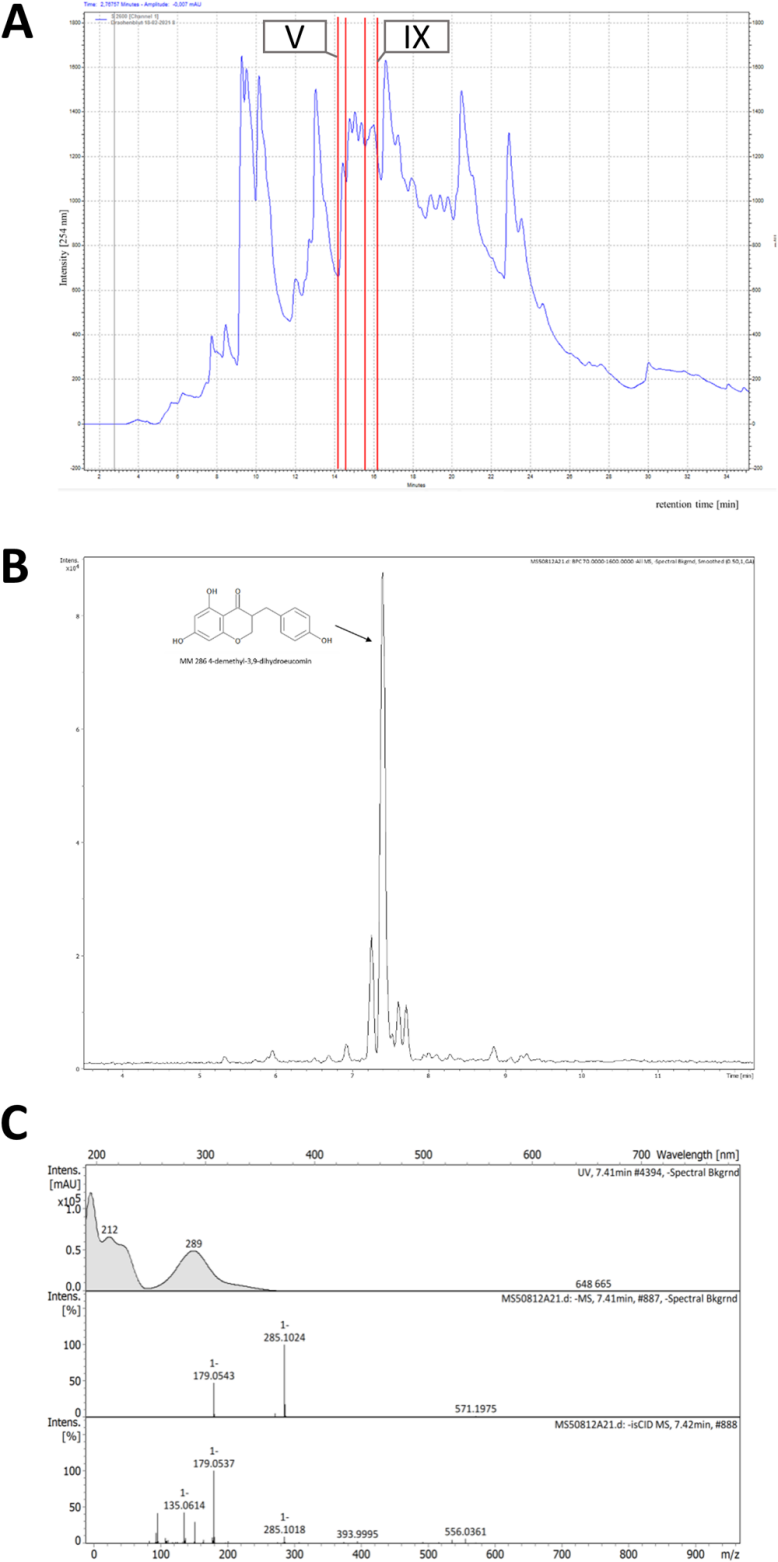
Preparative RP-18 chromatography of the DD extract (A), preparative silica gel chromatography of dragon blood sample **V**, and identification by means of LC-HRMS/CAS/UV and co-chromatography of compound 4′-demethyl-3,9-dihydroeucomin (**DMDHE**)([M – H]^−^ = 285.1026 *m*/*z*) (B, C).

For the identification of the key bitter-masking constituent, fractions **V** (yield: 172 mg), **IX** (yield: 313 mg), and **VI** (yield: 106 mg) were further separated by preparative RP-18 HPLC and analyzed by high-resolution mass spectrometry (LC-HRMS/CAD/UV, Figure 3B,C). The main component of sample faction **V** with 65 CAD-area percent and a molecular mass of 286 ([M – H]^−^ = 285.1026 *m*/*z*) was characterized by nuclear magnetic resonance (NMR) and identified as 2,3-dihydro-5,7-dihydroxy-3-[(4-hydroxyphenyl)methyl]-4*H*-1-benzopyran-4-one/5,7-dihydroxy-3-(4-hydroxybenzyl)chroman-4-one (**DMDHE**, mass 286 ([M – H]^−^ = 285.1026 *m*/*z*)), C_16_H_14_O_5_, trivial name 4′-demethyl-3,9-dihydroeucomin, ^1^H NMR (600 MHz, DMSO-*d*_6_) δ (ppm) 12.17 (s, 1H), 10.78 (s, 1H), 9,25 (s, 1H), 7.03–7.00 (m, 2H), 6.70–6.68 (m, 2H), 5.88 (d, *J* = 2.2 Hz, 1H), 5.86 (d, *J* = 2.1 Hz, 1H), 4.25 (dd, *J* = 11.4, 4.5 Hz, 1H), 4.07 (dd, *J* = 11.4, 8.1 Hz, 1H), 3.00 (dd, *J* = 13.9, 5.0 Hz, 1H), 2.95 (dddd, *J* = 9.5, 8.1, 5.0, 4.5 Hz, 1H), 2.58 (dd, *J* = 13.9, 9.5 Hz, 1H); ^13^C NMR (151 MHz, DMSO-*d*_6_) δ 197.8, 166.6, 163.8, 162.8, 155.9, 129.9 (2×), 128.0, 115.3 (2×), 101.3, 95.9, 94.7, 68.9, 45.6, 31.2, purity 65%.^27^

In fractions **IX** and **VI**, **DMDHE** was also identified with 9.4 and 16.1 CAD-area percents of the total peak area (LC-HRMS/CAD/UV), respectively (data not shown). Besides fractions **V**, **IX**, and **VI**, **DMDHE** was also detected in fraction **VII**. However, since these fractions did not show significant bitter-masking effects (Table 2), they were not included in the following experiments. Here, we hypothesize that **DMDHE**’s bitter-masking effect was not as strong as the putative counteracting effects of other constituents of these RP-18-HPLC fractions. Notably, fractions **III**, **IV**, and **VIII** also reduced the bitterness of quinine (Table 2) but were not included in the following approach, since **DMDHE** was not detected in these fractions.

### Chemical Synthesis of **DMDHE**

**Scheme 1:**
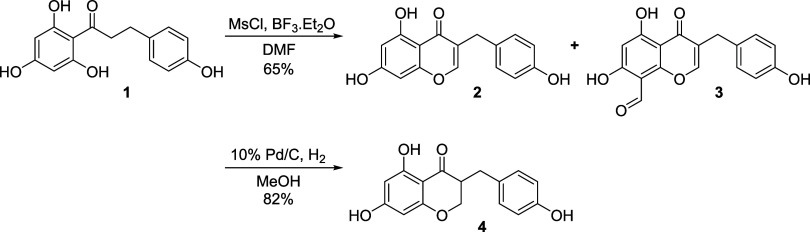
Gram-Scale Route to **DMDHE** (**4**); Access to Aldehyde **3**

Preparation of 5,7-dihydroxy-3-(4-hydroxybenzyl)-4*H*-chromen-4-one (**2**) and 5,7-dihydroxy-3-(4-hydroxybenzyl)-4-oxo-4*H*-chromene-8-carbaldehyde (**3**) was completed as follows (Scheme 1): To a stirred solution of phloretin **1** (8.00 g, 29.2 mmol) in DMF (50.0 mL) were added BF_3_·Et_2_O (5.60 mL, 43.8 mmol) and MsCl (2.71 mL, 35.0 mmol) dropwise at 0 °C. After the addition, the reaction mixture was heated to 80 °C and stirred for 2 h. The reaction was then cooled, diluted with cold water (50.0 mL), and the resulting mixture was extracted with EtOAc (100 mL × 3). The combined organic layer was washed with water and brine, then dried over anhydrous Na_2_SO_4_, and concentrated under reduced pressure. The residue was purified by silica gel column chromatography (eluent: EtOAc/hexane = 1/10 to 1/2), followed by trituration with DCM/hexane (1/3) to afford 5.38 g (65%) of pure chromen **2** as a pale-yellow powder and 65 mg of chromenaldehyde **3** as off-white powders.^28,29^

Preparation of 4′-demethyl-3,9-dihydroeucomin (**DMDHE**, **4**) was accomplished as follows: A mixture of chromen **2** (2.75 g, 0.97 mmol) in 50.0 mL of MeOH was added to 10% Pd/C (fresh and dry, 500 mg) under argon. The reaction was then degassed, charged with H_2_ (three times), and heated to 30 °C, stirring under H_2_ for 12 h. The reaction mixture was filtered through a pad of Celite, and the filtrate was concentrated. The residue was purified by silica gel column chromatography (eluent: DCM/MeOH = 50/1 to 30/1), followed by trituration with hexane and coevaporation with food-grade EtOH to afford 2.28 g (82%) of pure, off-white **DMDHE** (**4**). Spectroscopy data of ^1^H NMR, 13C-NMR, and MS are identical to the data of isolated **DMDHE** (see the Identification of **DMDHE** in DD Fractions section).

### Sensory Evaluation of Bitter-Masking Properties of **DMDHE**

**Figure 4:**
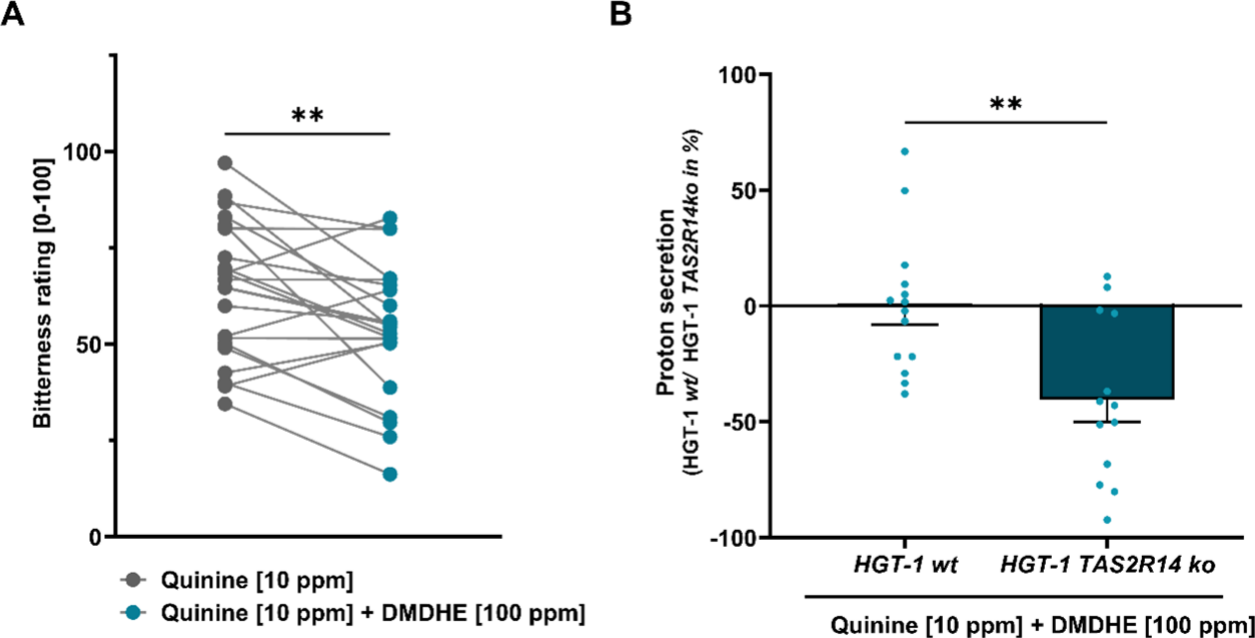
(A) Bitterness rating [0 not bitter–100 very bitter] of quinine [10 ppm] w/o addition of **DMDHE** [100 ppm] (*n* = 22). Statistical analysis: Paired Student’s *t*-test, significance is indicated with **(*p* ≤ 0.01). (B) Impact of quinine [10 ppm] + **DMDHE** [100 ppm] on the percent change in proton secretion in *TAS2R14* knockout cells versus HGT-1 wild-type cells (wt = 0%). Statistical analysis: Unpaired Student’s *t-*test, significance is indicated with **(*p* ≤ 0.001). Data are shown as mean ± SEM, *n* = 3, t.r. = 4–5.

The bitter-masking effect of **DMDHE** was evaluated by trained sensory panelists applying pairwise comparison tests to evaluate the bitterness of quinine [10 ppm] w/o **DMDHE** [100 ppm]. The mean reduction of quinine bitterness by **DMDHE** was reported at 14.8 ± 5.0%, reducing the quinine bitterness from 64.1 ± 3.7 to 53.8 ± 3.6 units on a scale from 0 (not bitter) to 100 (very bitter; Figure 4A; *p* < 0.01). Considering the sensory bitter-masking effect of DD, which was 29.6 ± 6.30% for a concentration of 500 ppm (Figure 2A), the here reported mean 14.8 ± 5.00% effect size for 100 ppm **DMDHE** can be considered stronger than that of the extract containing a number of compounds that presumably could act as TAS2R agonists/antagonists. Comparing the effect size of **DMDHE** with a recognized bitter-masking compound, the potential for its use in food formulations becomes apparent: Whereas a concentration of 100 ppm of HED reduced the bitterness of 5 ppm quinine by 31%,^30^ 100 ppm **DMDHE** decreased the bitter perception of 10 ppm quinine by 14%, demonstrating a similar effect size for **DMDHE** and HED.

### Elucidation of the Bitter-Masking Effect by **DMDHE** on a Molecular Level

When the bitter-masking effect of **DMDHE** on quinine was tested in the cellular bitter response assay, **DMDHE** [100 ppm] reduced the 10 ppm quinine-evoked proton secretion in HGT-1 cells by 296 ± 22.9% (data not shown). For the elucidation of the underlying molecular mechanisms, a CRISPR-Cas9 knockout of quinine-targeted *TAS2R14*(12) was performed. This broadly tuned TAS2R was chosen for further investigation, as its mRNA expression (i) is highest among all TAS2Rs in HGT-1 cells^9^ and (ii) was regulated by the addition of DD to quinine-treated cells (Table 1). Hence, functional involvement of *TAS2R14* was demonstrated by means of HGT-1 *TAS2R14* knockout cells, where the bitter-masking effect of **DMDHE** [100 ppm] on quinine [10 ppm], analyzed as percent proton secretion, was ameliorated by a mean of 40.4 ± 9.32% compared to HGT-1 wt cells (HGT-1 wt: 0.00 ± 7.74%, HGT-1 *TAS2R14ko*: −40.4 ± 9.32%; *p* < 0.01; Figure 4B).

With these results, we demonstrate another potential ligand of TAS2R14. From a chemical perspective, many of the TAS2R14 ligands share the structural property of a chroman ring, such as flavones, flavonols, flavanones, flavanonols, and flavanols.^16^ As an example, the TAS2R14 antagonist 6-methoxyflavanone^15^ as well as the TAS2R14 agonists homoeriodictyol (HED) and eriodictyol^16^ are characterized by the backbone of 2-phenylchromen-4-one (2-phenyl-1-benzopyran-4-one) as the core structure. This core structure is like that of **DMDHE**, which has an additional methyl group between the B- and C-rings. However, this structural modification of the C-ring might not be of utmost importance, at least. According to Roland et al.,^16^ the C-ring of flavans is hypothesized to be less critical for TAS2R activation than A- and B-rings.^16^ Most importantly, **DMDHE** and HED share the A-ring structure and a high similarity in the B-ring. Moreover, **DMDHE** shares an identical A- and B-ring structure with the potent TAS2R14 agonist naringenin, a bitter-tasting flavonoid of the flavone group.^13^ However, systematic molecular modeling studies are needed to clarify whether these structural elements are responsible for the bitter-masking properties of flavonoids. One also has to note that **DMDHE** is very likely not the only constituent that accounts for the strong bitter-masking effect of DD, as its yield therein was only 3%. However, the fact that the quantitatively dominating constituents of bitter-masking DD fraction **IX**, compounds **1** and **4** (Figures S1, S2, and S5), did not show any bitter-masking activity on quinine [10 ppm], supporting the potency of **DMDHE** as a minor constituent of DD identified by the activity-guided approach applied here.

The availability and identification of natural bitter-masking compounds still present major challenges for the food industry. Since bitter tastes can be a significant barrier to the acceptance, e.g., of plant-based foods containing naturally occurring bitter compounds, the so far limited number of bitter-maskers restricts the options of producing palatable food formulations. In this work, we identified **DMDHE** from the extract of the resin of *D. draco* as a promising novel bitter-masking compound, which might be suitable for reducing the bitterness of food and pharmaceutical formulations. For food applications, **DMDHE** holds the potential of a promising bitter-masking compound to be used in the context of clean labeling. Its advantage over the extract of the resin of *D. draco* lies in its use as an isolated, well-characterized plant compound, which is more reproducible and transparent to declare to the consumer than a plant extract.

## Supporting Information Available

Cell viability data of HGT-1 cells incubated with quinine and DMSO; extract of *D. draco* (DD) and **DMDHE** (Table S1); primer sequences used in qRT-PCR experiments (Table S2); genomic cleavage detection (Table S3); and preparative silica gel chromatogram of DD fraction **IX** (Figure S1) and structures of compounds therein (Figure S2–S5) (PDF)
