# Supplementary material for: Identification of 4′-Demethyl-3,9-dihydroeucomin as a Bitter-Masking Compound from the Resin of Daemonorops draco
Source: J Agric Food Chem. 2024 Sep 15;72(38):20991–9. doi: 10.1021/acs.jafc.4c04583 (PMC11440488; doi:10.1021/acs.jafc.4c04583)
Supplement: Supplementary file 1 — jf4c04583_si_001.pdf [file jf4c04583_si_001.pdf]

Supporting information to

Identification of 4'-Demethyl-3,9-Dihydroeucomin as a Bitter-Masking Compound from the Resin of *Daemonorops Draco*

Sonja Sterneder [1, 2, 3], Joachim Seitz [1], Johannes Kiefl [5], Eric Rottmann [5], Margit Liebig [5], Maria Blings [5], Stephan Seilwind [5], Yijun Zhou [6], Jianbing Wei [6], Haifeng Guan [6], Qianjin Zhu [6], Johanna Kreißl [3], Kai Lamottke [6, 7], Jakob P. Ley [5], Veronika Somoza [1, 3, 4]*

[1] Department of Physiological Chemistry, Faculty of Chemistry, University of Vienna, 1090 Vienna, Austria

[2] Vienna Doctoral School in Chemistry (DoSChem), Faculty of Chemistry, University of Vienna, 1090 Vienna, Austria

[3] Leibniz Institute for Food Systems Biology, Technical University of Munich, 85354 Freising, Germany

[4] Chair of Nutritional Systems Biology, TUM School of Life Sciences, Technical University of Munich, 85354 Freising, Germany

[5] Symrise AG, 37603 Holzminden, Germany

[6] Bicoll Biotechnology (Shanghai) Co. Ltd., 201203 Pudong, China

[7] Bicoll GmbH, 82152 Planegg/Martinsried, Germany

* Corresponding author: Email: v.somoza.leibniz-lsb@tum.de. Phone: +49-8161-71-2700.

Tables

Table S1: Cell viability of HGT-1 cells incubated with quinine and DMSO (0.1%), the extract of Daemonorops draco (DD) and DMDHE for 15 minutes. Data shown in mean percent of treated over control (KRHB-treated cells = 100 %) ± SEM. Statistics: One Way ANOVA with post-hoc Kruskal-Wallis test for comparison between control and treated cells revealed no statistically significant difference, n = 3-6, t.r.= 2-3.

**Incubation**		**Cell viability [%]**
KRHB (control)		100 ± 2.24
KRHB +	DMSO [0.1%]	110 ± 5.87
	DD [100 ppm]	110 ± 4.35
	DMDHE [100 ppm]	112 ± 4.32
Quinine [10 ppm]		108 ± 4.71
Quinine [10 ppm] +	DMSO [0.1%]	104 ± 3.84
	DD [100 ppm]	114 ± 1.96
	DMDHE [100 ppm]	113 ± 3.21

Table S2: Primer sequences used in qRT-PCR. [9]

Gene	Direction	Sequence 5'-3'	bp
GAPDH	**FW**	AGGTCGGAGTCAACGGATTTG	95
	**RV**	GGGGTCATTGATGGCAACAATA	
PPIA	**FW**	CCACCAGATCATTCCTTCTGTAGC	144
	**RV**	CTGCAATCCAGCTAGGCATGG	
TBP	**FW**	CCCGAAACGCCGAATATAATC	130
	**RV**	GACTGTTCTTCACTCTTGGCTC	
TAS2R4	**FW**	GCAGTGTCTGGTTTGTGACC	168
	**RV**	GCGTGATGTACAGGCAAGTG	
TAS2R7	**FW**	CCCTGCGGAGACATATCAGG	91
	**RV**	ACAGCTTTCAGGGCTCTCAC	
TAS2R10	**FW**	GCTACGTGTAGTGGAAGGCA	73
	**RV**	TCCATTCCCCAAAACCCCAA	
TAS2R14	**FW**	CCAGGTGATGGGAATGGCTTA	128
	**RV**	AGGGCTCCCCATCTTTGAAC	
TAS2R31	**FW**	TTGAGGAGTGCAGTGTACCTTTC	218
	**RV**	ACGGCACATAACAAGAGGAAAA	
TAS2R39	**FW**	TTCTGTGGCTGTCCGTGTTTA	207
	**RV**	GGGTGGCTGTCAGGATGAAC	
TAS2R40	**FW**	CGGTGAACACAGATGCCACAGATA	150
	**RV**	GTGTTTTGCCCCTGGCCCACT	
TAS2R43	**FW**	ATATCTGGGCAGTGATCAACC	148
	**RV**	CCCAACAACATCACCAGAATGAC	
TAS2R46	**FW**	ACATGACTTGGAAGATCAAACTGAG	72
	**RV**	AGTTTGCTAGGATGGTTACCGTT	

Table S3: Primer sequences used for genomic cleavage detection.

**Gene**	**Direction**	**Sequence 5‘-3‘**	**bp**
HPRT1	**FW**	ACA TCA GCA GCT GTT CTG	445
	**RV**	GGC TGA AAG GAG AGA ACT	
TAS2R14	**FW**	GCAGCACCAAAACCACCTTT	396
	**RV**	GGCAATGGGTGGTGTCATAA	

SI - Figures

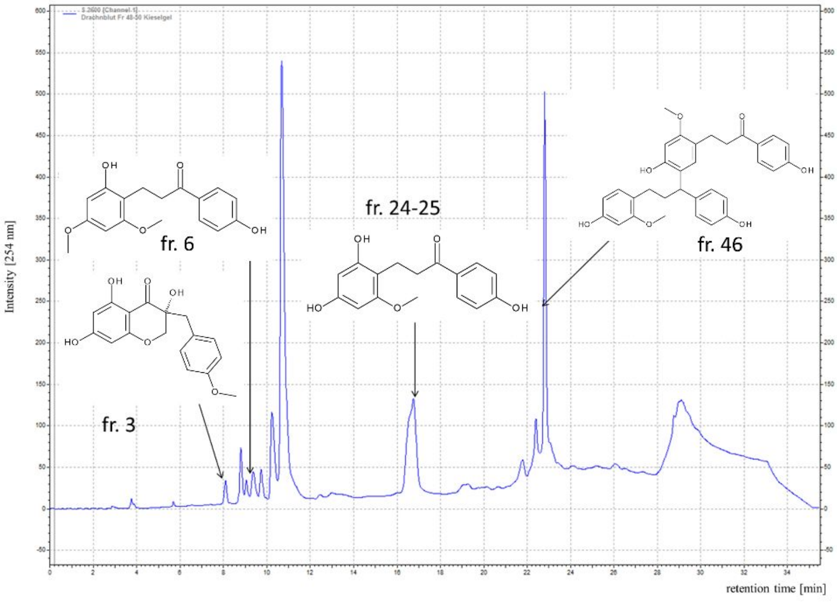

Figure S1: Preparative silica gel chromatography of DD fractions **IX**. Fractions were collected peak by peak and only the fractions with the highest yield (fraction 3 with 6.7 mg, 5 with 27.5 mg, 6 with 14.5 mg, 24-25 with 8.3 mg and 46 with 12.1 mg) were considered for further evaluations. Compound **DMDHE** and compound **1**-**4** were identified as constituents of these fractions by LC-HRMS and NMR at room temperature in fraction 3, 6, 24-25 and 46. Compound 1 and 4 were tested on their bitter masking activity on quinine in human sensory analysis and did not show any effect (data not shown).

Compound 1:

2,3-dihydro-3,5,7-trihydroxy-3-[(4-methoxyphenyl)methyl]-4H-1-Benzopyran-4-one (Fraction **IX** subfraction 3, mass 316, C17H16O6, purity 98%, trivial name Eucomol): 1H NMR (400 MHz, DMSO) δ (ppm) 11.95 (s, 1H), 7.19 – 7.11 (m, 2H), 6.88 – 6.81 (m, 2H), 5.99 (s, 1H), 5.92 (d, *J* = 2.1 Hz, 1H), 5.90 (d, *J* =2.1 Hz, 1H), 3.97 (d, *J* = 11.4 Hz, 1H), 3.93 (d, *J* = 11.4 Hz, 1H), 3.73 (s, 3H), 2.86 (s, 2H). 13C NMR (101 MHz, DMSO) δ (ppm) 198.3, 166. 9, 164.0, 162.6, 158.1, 131.6, 127.0, 113.4, 100.0, 96.2, 95.0, 71.6, 71.6, 55.0, 38.5.

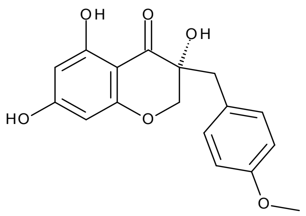

Figure S2: Compound 1 Eucomol

Compound 2:

3-(2-Hydroxy-4,6-dimethoxyphenyl)-1-(4-hydroxyphenyl)-1-propanone (Fraction **IX** subfraction fr. 6, mass 302, C17H18O5, purity 80%); 1H NMR (400 MHz, DMSO) δ (ppm) 9.35 (s, 1H), 7.87 –7.82 (m, 2H), 6.86 – 6.80 (m, 2H), 6.06 (d, *J* = 2.4 Hz,1H), 6.05 (d, *J* = 2.4 Hz, 1H), 3.71 (s, 3H), 3.68 (s,3H), 2.95 – 2.88 (m, 2H), 2.78 – 2.70 (m, 2H); 13C NMR (101 MHz, DMSO) δ (ppm) 198.3, 161.9, 158.8, 158.7, 156.2, 130.5, 128.1, 115.2, 107.4, 93.6, 89.8, 55.4, 54.9, 37.8, 18.4.

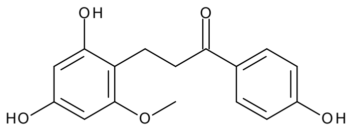

Figure S3: Compound 2 3-(2-Hydroxy-4,6-dimethoxyphenyl)-1-(4-hydroxyphenyl)-1-propanone

Compound 3:

3-(2,4-dihydroxy-6-methoxyphenyl)-1-(4-hydroxyphenyl)-1-propanone (Fraction **IX** subfraction fr. 24-25, mass 288, C16H16O5, purity 95%, trivial name Loureirin D); 1H NMR (400 MHz, DMSO) δ (ppm) 10.30 (s, 1H), 9.14 (s, 1H), 9.04 (s, 1H), 7.90 – 7.77 (m, 2H), 6.88 – 6.78 (m, 2H), 5.96 (d, *J* = 2.1 Hz, 1H), 5.88 (d, *J* = 2.1 Hz, 1H), 3.65 (s, 3H), 2.93 –2.85 (m, 2H), 2.75 – 2.64 (m, 2H); 13C NMR (101 MHz, DMSO) δ (ppm) 198.5, 161.9, 158.7, 156.6, 156.2, 130.5, 128.2, 115.2, 105.7, 95.4, 90.7, 55.2, 38.1, 18.5.

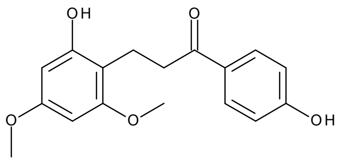

Figure S4: Compound 3 Loureirin D

Compound4:

3-[4-hydroxy-5-[3-(4-hydroxy-2-methoxyphenyl)-1-(4-hydroxyphenyl)propyl]-2-methoxyphenyl]-1-(4-hydroxyphenyl)-1-propanone (Fraction **IX** subfraction fr. 46, mass 528, C32H32O7, CAS 909250-39-1, purity 90%); ^{1}H NMR (400 MHz, DMSO) δ (ppm) 10.31 (s, 1H), 9.14 (s, 1H), 9.05 (s, 1H), 9.04 (s, 1H), 7.85 –7.78 (m, 2H), 7.01 – 6.96 (m, 2H), 6.96 (s, 1H),6.86 – 6.81 (m, 2H), 6.79 (d, *J* = 8.1 Hz, 1H), 6.62 – 6.58 (m, 2H), 6.36 (s, 1H), 6.33 (d, *J* =2.3 Hz, 1H), 6.24 (dd, *J* = 8.1, 2.3 Hz, 1H), 4.05 (t, *J* = 7.8 Hz, 1H), 3.68 (s, 3H), 3.67 (s, 3H), 3.05 (t, *J* = 7.6 Hz, 2H), 2.84 – 2.68 (m, 2H), 2.36 – 2.20 (m, 2H), 2.08 – 1.91 (m, 2H); ^{13}C NMR (101 MHz, DMSO) δ (ppm) 198.0, 161.9, 157.8, 156.6, 155.4, 155.0, 153.6, 136.1, 130.4, 129.6, 128.6, 128.4, 128.2, 123.0, 120.4, 118.9, 115.2, 114.7, 106.5, 98.9, 98.6, 55.1, 55.0, 41.22, 38.3, 35.45, 27.8, 24.9.

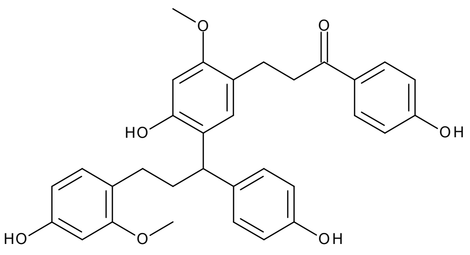

Figure S5: Compound 4 3-[4-hydroxy-5-[3-(4-hydroxy-2-methoxyphenyl)-1-(4-hydroxyphenyl) propyl]-2-methoxyphenyl]-1-(4-hydroxyphenyl)-1-propanone
